# Oligomerization and Spatial Distribution of Kvβ1.1 and Kvβ2.1 Regulatory Subunits

**DOI:** 10.3389/fphys.2022.930769

**Published:** 2022-06-17

**Authors:** Sara R. Roig, Silvia Cassinelli, Andre Zeug, Evgeni Ponimaskin, Antonio Felipe

**Affiliations:** ^1^ Molecular Physiology Laboratory, Departament de Bioquímica i Biomedicina Molecular, Institut de Biomedicina (IBUB), Universitat de Barcelona, Barcelona, Spain; ^2^ Imaging Core Facility, Biozentrum, University of Basel, Basel, Switzerland; ^3^ Department of Cellular Neurophysiology, Hannover Medical School, Hannover, Germany

**Keywords:** regulatory subunits, oligomerization, potassium channels, lipid rafts, leukocytes

## Abstract

Members of the regulatory Kvβ family modulate the kinetics and traffic of voltage-dependent K^+^ (Kv) channels. The crystal structure of Kv channels associated with Kvβ peptides suggests a α4/β4 composition. Although Kvβ2 and Kvβ1 form heteromers, evidence supports that only Kvβ2.1 forms tetramers in the absence of α subunits. Therefore, the stoichiometry of the Kvβ oligomers fine-tunes the activity of hetero-oligomeric Kv channel complexes. We demonstrate that Kvβ subtypes form homo- and heterotetramers with similar affinities. The Kvβ1.1/Kvβ2.1 heteromer showed an altered spatial distribution in lipid rafts, recapitulating the Kvβ1.1 pattern. Because Kvβ2 is an active partner of the Kv1.3-TCR complex at the immunological synapse (IS), an association with Kvβ1 would alter this location, shaping the immune response. Differential regulation of Kvβs influences the traffic and architecture of the Kvβ heterotetramer, modulating Kvβ-dependent physiological responses.

## Introduction

The association of α-conducting subunits with β regulatory peptides determines the functional diversity of voltage-gated potassium (Kv) currents. Thus, changes in the expression of a subunit shape the channel composition and the physiological properties of the channelosome (Pongs and Schwarz, 2010).

Three genes (Kvβ1-3) encode the Kvβ family, some undergoing alternative splicing (i.e., Kvβ1.1–Kvβ1.3). Kvβs exhibit 85% similarity, mostly at the C-terminus. However, several functional differences, focused on Kv modulation, have been described (Kilfoil et al., 2013). While Kvβ1 and Kvβ3 accelerate the fast inactivation of Kv channels, using a ball-and-chain mechanism (Heinemann et al., 1995; Leicher et al., 1996), Kvβ2 increases the surface expression of the complex (Shi et al., 1996). Kvβ peptides exhibit aldo-keto reductase (AKR) activity by binding to NADP(H) and are included in the AKR6A subfamily within the AKR superfamily (Hyndman et al., 2003). Biochemical and structural evidence confirmed the presence of more than one Kvβ subunit in a tetrameric Kv channel configuration (Parcej et al., 1992; Gulbis et al., 1999). Scarce information is available regarding the oligomeric formation of Kvβ peptides. Evidence suggests that Kvβ1 and Kvβ2 form homo and hetero-oligomeric compositions (Xu et al., 1998; Nystoriak et al., 2017). Atomic force and electron microscopy support that the complex architecture is a tetramer with possible intermediate structures (van Huizen et al., 1999). The Kvβ2 crystal determines not only the macromolecular structure but also the orientation of units (Gulbis et al., 1999).

The molecular determinants for Kvβ2 oligomeric formation are mainly located within the core region. Kvβ1.1 and Kvβ2.1 share most of the C-terminal sequence, but Kvβ1.1 presents no interacting domains. Regarding the function of Kv, evidence shows that the higher the expression of Kvβ1 is, the larger the inactivation rate. Therefore, the variable stoichiometry of α_4_β_n_ (4 α-subunits with a flexible number of β-subunits) would exert important physiological consequences (Xu et al., 1998). Thus, Kvβ2 inhibits the Kvβ1-mediated inactivation of Kv channels. This effect requires the core part of the subunit, which is necessary not only for homo- and hetero-oligomerization but also for interaction with the channel. Kvβ2 interacts with Kv channels in a tetrameric structure displacing Kvβ1, leading to a nonconcentration dependence of Kv modulation (Xu and Li, 1997). However, similar to Kvβ2, the capacity of Kvβ1.2 to form homo-oligomers has also been documented. Therefore, hybrids of both proteins with Kv channels induce intermediate inactivation patterns on Kv1.2 (Accili et al., 1997). This function could be of special relevance in immune system physiology, where Kvβ peptides are tightly regulated under insults (McCormack et al., 1999; Vicente et al., 2005). Kvβ2 concentrates with Kv1.3 at the immunological synapse (IS) (Beeton et al., 2006; Roig et al., 2022). Furthermore, Kvβ2, located in lipid rafts, may cluster in these spots, independent of the channel, during the immune response (Roig et al., 2022). Unlike Kvβ1, this spatial regulation is dependent on palmitoylation. The fact that Kvβ1.1 may alter the Kvβ2 spatial location at the IS, influencing the Kv1.3-dependent physiological consequences, could be crucial during the immune response.

Evidence suggest that Kvβ peptides could have physiological functions, such as REDOX sensors and clustering targeting proteins to specific spots, without the participation of Kv α subunits (Beeton et al., 2006; Roig et al., 2022). In this context, our results demonstrated that Kvβ1.1, as well as Kvβ2.1, are tetramers. In addition, we found that the affinity to form Kvβ homo- and hetero-oligomers is similar. Both Kvβ subunits reach the cell surface in homo- and heteromeric forms but with different plasma membrane distributions. While Kvβ2.1 partially targets lipid rafts, the combination with Kvβ1.1 mistargeted these domains. Therefore, Kvβ1, whose abundance is under tight regulation, would fine-tune the final fate and stoichiometry of the functional Kvβ complex, thereby shaping Kv1.3-dependent physiological responses.

## Materials and Methods

### Expression Plasmids, Cell Culture and Transfections

mKvβ1.1 and mKvβ2.1 were provided by M.M. Tamkun (Colorado State University). mKvβ1.1 and mKvβ2.1 were subcloned into pEYFP-N1 and pECFP-N1 (Clontech). All constructs were verified by sequencing.

HEK-293 cells were cultured in DMEM (Lonza) containing 10% fetal bovine serum (FBS) supplemented with penicillin (10,000 U/ml), streptomycin (100 μg/ml) and L-glutamine (4 mM) (Gibco). Human Jurkat T-lymphocytes and the murine CY15 dendritic cell line were cultured in RPMI culture medium (Lonza) containing 10% heat-inactivated FBS and supplemented with 10,000 U/ml penicillin, 100 µg/ml streptomycin and 2 mM L-glutamine (Gibco). Human CD4^+^ T-cell subsets were isolated from peripheral whole blood using a negative selection Rosette Sep^TM^ kit from STEMCELL^TM^ Technologies. Human T lymphocytes were cultured as previously described (Capera et al., 2021). Murine bone marrow-derived macrophages (BMDMs) from 6- to 10-week-old BALB/c mice (Charles River Laboratories) were used. The cells were isolated and cultured as described elsewhere (Sole et al., 2013).

For confocal imaging and coimmunoprecipitation experiments, cells were seeded (70–80% confluence) in 6-well dishes containing polylysine-coated coverslips or 100 mm dishes 24 h before transfection with selected cDNAs. Lipotransfectina® (Attendbio Research) was used for transfection according to the supplier’s instructions. The amount of transfected DNA was 4 µg for a 100 mm dish and 500 ng for each well of a 6-well dish (for coverslip use). Next, 4–6 h after transfection, the mixture was removed from the dishes and replaced with fresh culture medium. All experiments were performed 24 h after transfection.

### Protein Extraction, Coimmunoprecipitation and Western Blotting

All experimental protocols were approved by the ethical committee of the Universitat de Barcelona in accordance with the European Community Council Directive 86/609 EEC. We also confirm that all experiments were carried out in compliance with the ARRIVE guidelines (https://arriveguidelines.org). Rats and mice were briefly anesthetized with isoflurane, and brains and femurs were extracted immediately after euthanasia. The brain was homogenized in RIPA lysis buffer (1% Triton X-100, 1% sodium deoxycholate, 0.1% SDS, 50 mM Tris-HCl pH 8.0, 150 mM NaCl) supplemented with protease inhibitors. Total lysates were spun for 10 min at ×10,000 g to remove debris. Supernatants were used to analyze protein expression by western blotting.

Cells were washed twice in cold PBS and lysed on ice with lysis buffer (5 mM HEPES, 150 mM NaCl, 1% Triton X100, pH 7.5) supplemented with 1 μg/ml aprotinin, 1 μg/ml leupeptin, 1 μg/ml pepstatin and 1 mM phenylmethylsulfonyl fluoride as protease inhibitors. Cells were scraped and transferred to a 1.5 ml tube. Then, they were incubated for 20 min at the orbital at 4°C and spun for 20 min at 14,000 rpm. The supernatant was transferred to a new tube, and the protein contents were determined by using the Bio–Rad Protein Assay (Bio–Rad).

For coimmunoprecipitation, 1 mg of protein from each condition was separated and brought up to a volume of 500 μL with lysis buffer for IPs (150 mM NaCl, 50 mM HEPES, 1% Triton X-100, pH 7.4) supplemented with protease inhibitors. Precleaning was performed with 40 μL of protein A Sepharose beads (GE Healthcare) in an orbital shaker for 1 h at 4°C. Next, each sample was incubated in a small chromatography column (Bio–Rad Microspin Chromatography Columns), which contained 2.5 μg of anti-GFP antibody (Genescript) previously crosslinked to protein A Sepharose beads, for 2 h at room temperature (RT) with continuous mixing. Next, columns were centrifuged for 30 s at ×1,000 g. The supernatant (SN) was kept and stored at −20°C. The columns were washed four times with 500 μL of lysis buffer and centrifuged for 30 s at ×1,000 g. Finally, elution was performed by incubating the columns with 100 μL of 0.2 M glycine pH 2.5 and spun 30 s at ×1,000 g. The eluted proteins (IP) were prepared for western blotting by adding 20 μL of loading buffer (×5) and 5 μL of 1 M Tris-HCl pH 10.

Irreversible crosslinking of the antibody to the Sepharose beads was performed after 1 h of incubation at RT of the antibody with protein A Sepharose beads, incubating the beads with 500 μL of dimethyl pimelimidate (DMP, Pierce) for 30 min at RT. Next, the columns were washed four times with 500 μL of ×1 TBS, four times with 500 μL of 0.2 M glycine pH 2.5 and three times more with ×1 TBS. Next, the columns were incubated with the protein lysates to perform immunoprecipitation following the above-described protocol.

Protein samples (50 μg), SN and IP were boiled in Laemmli SDS loading buffer and separated by 10% SDS–PAGE. For the nondenaturing technique, no boiling step was applied, and the SDS–PAGE gel was 8%. Next, samples were transferred to nitrocellulose membranes (Immobilon-P, Millipore) and blocked in 0.2% Tween-20-PBS supplemented with 5% dry milk before immunoreaction. Filters were immunoblotted with antibodies against Kvβ1.1 (1/1,000, Neuromab), Kvβ2.1 (1/1,000, Neuromab), Clathrin (1/1,000, BD Transduction) or Caveolin (1/1,000, BD transduction). Finally, the filters were washed with 0.05% Tween 20 PBS and incubated with horseradish peroxidase-conjugated secondary antibodies (Bio–Rad).

### Confocal Microscopy and Image Analysis

For confocal microscopy, cells were seeded on poly-lysine-coated coverslips and transfected 24 h later. The next day, the cells were quickly washed twice, fixed with 4% paraformaldehyde for 10 min, and washed three times for 5 min with PBS-K+. Finally, coverslips were mounted on microscope slides (Acefesa) with house Mowiol mounting media. Coverslips were dried at RT at least 1 day before imaging.

The fluorescence resonance energy transfer (FRET) via the acceptor photobleaching technique was measured in a discrete region of interest (ROI). Fluorescent proteins from fixed cells were excited with the 458 nm or the 514 nm lines using low excitation intensities. Next, 475–495 nm bandpass and >530 nm longpass emission filters were applied. The YFP was bleached using maximum laser power with a yield of approximately 80% acceptor bleaching. After photobleaching of the acceptor, images of the donors and acceptors were captured. The FRET efficiency was calculated using the equation.

[(F_CFPafter_–F_CFPbefore_)/F_CFPafter_]×100, where F_CFPafter_ and F_CFPbefore_ are the fluorescence of the donor after and before bleaching, respectively. The loss of fluorescence as a result of the scans was corrected by measuring the CFP intensity in the unbleached part of the cell. All images were acquired with a Leica TCS SL laser scanning confocal spectral microscope (Leica Microsystems) equipped with argon and helium–neon lasers. All experiments were performed with a ×63 oil-immersion objective lens NA 1.32. All offline image analyses were performed using ImageJ software.

### Cell Unroofing Preparations

HEK-293 cells were seeded in poly-D-lysine-treated glass coverslips. Twenty-four hours after transfection, they were cooled on ice for 5 min and washed twice in PBS without K^+^. Next, the samples were incubated for 5 min in KHMgE buffer (70 mM KCl, 30 mM HEPES, 5 mM MgCl_2_, 3 mM EGTA, pH 7.5) diluted three times and then gently washed with nondiluted KHMgE to induce hypotonic shock. Burst cells were removed from the coverslip by intensively pipetting up and down. After two washes with KHMgE buffer, only membrane sheets remained attached. Preparations were fixed and mounted as previously described (Oliveras et al., 2020).

### Lipid Raft Isolation

Low density Triton-insoluble complexes were isolated as previously described (Martinez-Marmol et al., 2008) from HEK-293 cells transiently transfected with Kvβ1.1CFP and Kvβ2.1CFP. Cells were homogenized in 1 ml of 1% Triton X-100, and sucrose was added to a final concentration of 40%. A 5–30% linear sucrose gradient was layered on top and further centrifuged (×390,000 g) for 20–22 h at 4°C in a Beckman SW41Ti rotor. Gradient fractions (1 ml) were sequentially collected from the top and analyzed by western blotting.

### Spectral Lux-Fluorescence Resonance Energy Transfer Analysis in Living Cells

Linear unmixing FRET (lux-FRET) described in (Wlodarczyk et al., 2008; Prasad et al., 2013) is a quantitative spectral FRET approach, based on two excitations, preferentially but not necessary where donor and acceptor are best excited, respectively. Lux-FRET treats the variety of possible distances, i.e., FRET states, of donor and acceptor as superposition of free donor and acceptor, and DA complexes. Since lux-FRET is based on spectral unmixing, references of donor only and acceptor only are required. Furthermore, like in other spectral FRET approaches, lux-FRET requires one tandem construct with a fixed one-to-one stoichiometry of donor and acceptor fluorophore. Because the calibration of the tandem construct is independent of the FRET efficiency, the information about the FRET efficiency of the tandem construct is not necessary. With the knowledge of the fluorescence quantum yield of the donor and acceptor fluorophores, lux-FRET is able to deduce the apparent FRET efficiencies Ef_D_ and Ef_A_, where $f_{D}=\frac{\left[DA\right]}{\left[D^{t}\right]}$ and $f_{A}=\frac{\left[DA\right]}{\left[A^{t}\right]}$ are the fractions of donors and acceptors in complexes, respectively; the donor molar fraction ( $x_{D}=\frac{\left[D^{t}\right]}{\left[D^{t}\right]+\left[A^{t}\right]}$ ); and the total donor (D) and acceptor (A) quantities, [D^t^] and [A^t^], scaled to the reference concentrations. For that, HEK-293 cells were cotransfected with Kvβ1.1YFP and Kvβ2.1CFP. Twenty-four hours after transfection, the cells were resuspended in PBS. All lux-FRET measurements were recorded with the fluorescence spectrometer Fluorog-3.22 (Horiba) equipped with a xenon lamp (450 W, 950 V) and two double monochromators. Following configuration and settings were used: 5-mm pathway quartz cuvettes at 37°C in “front face” arrangement, dual excitation 440 and 488 nm, with emission spectra 450–600 nm and 500–600 nm, respectively, 0.5 s integration time. The spectral contributions from light scattering and nonspecific fluorescence of the cells were taken into account by subtracting the emission spectra of non-transfected cells (background) from each measured spectrum. Before the measurements, the spectrofluorometer was calibrated for the xenon lamp spectrum and Raman scattering peak position. To determine the apparent FRET efficiency for Kvβ1.1 and Kvβ2.1, we used a method described in detail previously (Renner et al., 2012; Prasad et al., 2013)). In short, we obtained relative excitation strengths r^ex,1^ and r^ex,2^ of the donor and acceptor from cells expressing, e.g., Kvβ1.1CFP or Kvβ2.1YFP only and did a non-negative linear unmixing with the corresponding characteristic, the Mock and the Raman spectrum. In the same way we received the donor and acceptor contributions δ^i^ and α^i^, for both excitations i from co-expressions of donor and acceptor of various relative expression levels. The relative experimental donor to acceptor brightness $R_{TC}=\frac{\alpha_{TC}^{1}\cdot r^{ex,2}-\alpha_{TC}^{2}\cdot r^{ex,1}}{\Delta r\cdot \delta_{TC}^{1}+\Delta \alpha_{TC}}$ , with $\Delta r=r^{ex,2}-r^{ex,1}$ and $\Delta \alpha =\alpha^{2}-\alpha^{1}$ , required for further calculations, we obtained from a tandem construct TC with one-to-one stoichiometry of donor and acceptor. From that, we calculated the total concentration ratio $\left[A^{t}\right]/\left[D^{t}\right]=\frac{\alpha^{1}\cdot r^{ex,2}-\alpha^{2}\cdot r^{ex,1}}{R_{TC}\cdot \left(\Delta r\cdot \delta^{1}+\Delta \alpha \right)}$ of the donor and acceptor, the donor molar fraction (x_D_) $x_{D}=\frac{\left[D^{t}\right]}{\left[D^{t}\right]+\left[A^{t}\right]}=1/\frac{1}{1+\left[A^{t}\right]/\left[D^{t}\right]}$ and the apparent FRET efficiencies $Ef_{D}=\frac{\Delta \alpha }{\Delta r\cdot \delta^{1}+\Delta \alpha }$ and $Ef_{A}=R_{TC}\cdot \frac{\Delta \alpha }{\alpha^{1}\cdot r^{ex,2}-\alpha^{2}\cdot r^{ex,1}}$ (Wlodarczyk et al., 2008). The model characterizing apparent FRET efficiency (Ef_D_) as a function of donor mole fraction (x_D_) for oligomeric structures was developed previously (Veatch and Stryer, 1977) following $Ef_{D}=E\left(1-x_{D}^{n-1}\right)$ . Fitting this model to experimental data yields the true transfer efficiency (E) and provides information about the number of units (n) interacting within the oligomeric complex. This model was slightly augmented for use with $Ef_{A}=E\frac{x_{D}}{x_{D}-1}\left(1-x_{D}^{n-1}\right)$ (Meyer et al., 2006). The oligomerization model, yielding the total FRET efficiency, the basic subunit formation and the affinity constants, was previously described (Renner et al., 2012).

## Results

### Kvβ1.1 and Kvβ2.1 are able to Homo- and Heteroligomerize

**FIGURE 1:**
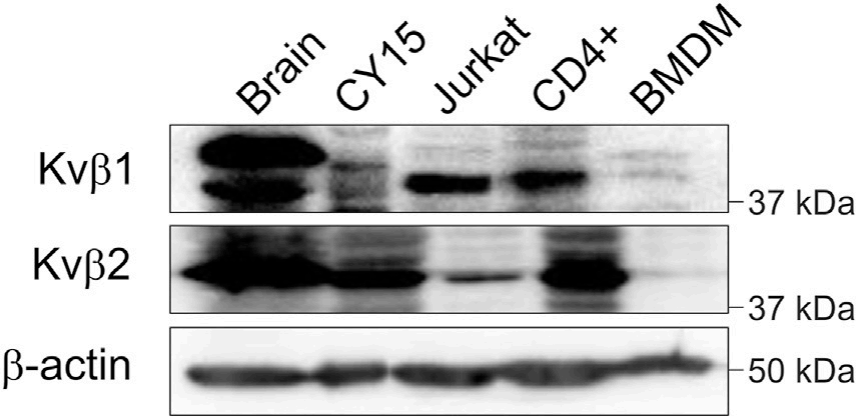
Kvβ1 and Kvβ2 expression in the brain and leukocytes. Total lysates from rat brain and different leukocytes were immunoblotted against Kvβ1 and Kvβ2. Mouse dendritic CY15, human Jurkat T cells, human CD4+ lymphocytes, and murine BMDM samples were obtained and processed as described in the methods. Top panel, Kvβ1; middle panel, Kvβ2; bottom panel, β-actin as a control. Note that representative western blots are shown only for qualitative purposes. No comparison among groups due to species, cell type and sample processing is intended.

The nervous and immune systems express members of the voltage-gated regulatory subunit family Kvβ (McCormack et al., 1999; Vicente et al., 2005; Pongs and Schwarz, 2010). Kvβ1 and Kvβ2 are involved in controlling Kv inactivation and spatial distribution, such as axonal targeting and IS location (Gu et al., 2003; Beeton et al., 2006; Roig et al., 2022). Therefore, we confirmed that the brain and leukocytes expressed the Kvβ1 and Kvβ2 subunits. As expected, not only the rat brain but also a wide repertoire of leukocytes, such as mouse CY15 dendritic cells, human Jurkat T cells, human CD4^+^ lymphocytes and primary murine bone marrow-derived macrophages (BMDMs), expressed both Kvβ1 and Kvβ2 regulatory subunits (Figure 1). Therefore, Kvβs are ubiquitously expressed within the immune system.

**FIGURE 2:**
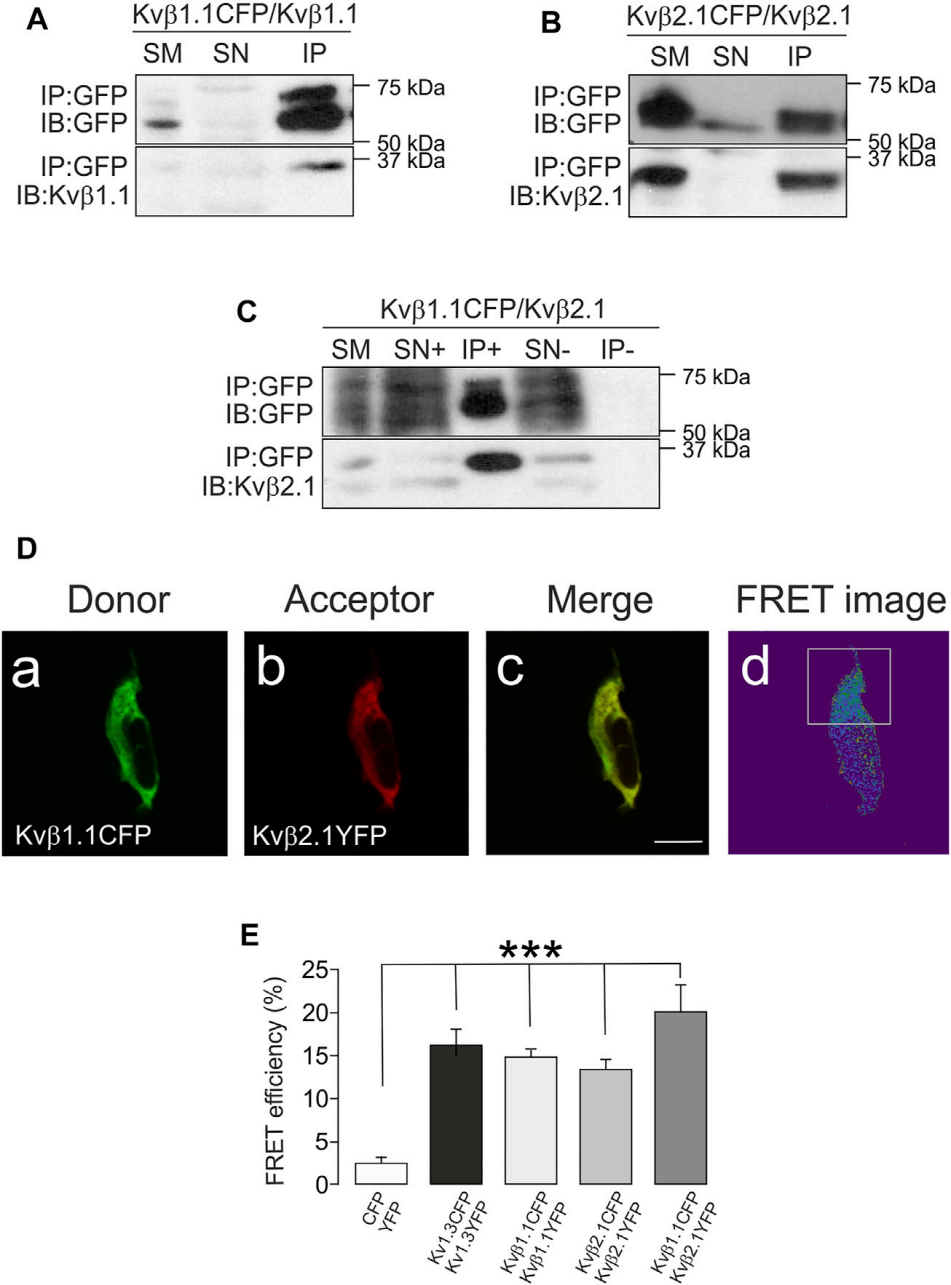
Kvβ1.1 and Kvβ2.1 form oligomers. HEK 293 cells were transfected with Kvβ1.1 and Kvβ2.1. **(A)** Coimmunoprecipitation assay against Kvβ1.1CFP in the presence of Kvβ1.1. **(B)** Coimmunoprecipitation assay against Kvβ2.1CFP in the presence of Kvβ2.1. **(C)** Coimmunoprecipitation assay against Kvβ1.1CFP in the presence of Kvβ2.1. **(C)**. Top panels: immunoprecipitation (IP) of CFP and immunoblot (IB) against CFP. Bottom panels: IB against Kvβ1.1 **(A)** and Kvβ2.1 **(B**,**C)**. SM: starting material. SN+: supernatant in the presence of the antibody. IP+: Immunoprecipitation in the presence of the antibody. SN-: supernatant in the absence of the antibody. IP-: immunoprecipitation in the absence of the antibody. **(D)** Kvβ1.1 and Kvβ2.1 form homo- and hetero-oligomers. Representative image of Kvβ1.1 and Kvβ2.1 cotransfection. **(Da)** Donor, Kvb1.1CFP; **(Db)** Acceptor, Kvb2.1 YFP; **(Dc)** merge, yellow indicates colocalization; **(Dd)** FRET image obtained from the relationship between the donor prebleach versus postbleach after acceptor photobleaching. The white square highlights the bleached section. The bar represents 20 μm. **(E)** FRET efficiencies (%) calculated from the acceptor photobleaching experiments. Values are the mean ± SE of >30 cells. ***p < 0.01 vs. CFP/YFP negative control (Student’s t test).

Most of the work related to the Kvβ family addresses the regulation of Kv channels (Pongs and Schwarz, 2010). Some studies address the modulation, *via* AKR activity, of the α-subunits. However, scarce information is available on the putative oligomeric formation of Kvβs. Evidence indicates that Kvβ2, but not Kvβ1, forms complexes. Kvβ1 controls channel activity in a concentration-dependent manner, and Kvβ2, by trapping Kvβ1 in those complexes, could impair its function on the α-subunits (Xu and Li, 1997; Xu et al., 1998). Structural studies indicate a prevalent tetrameric composition for the Kvβ2 complexes (Gulbis et al., 1999; van Huizen et al., 1999). Although evidence demonstrates that Kvβ2.1 forms homo- and heteromers with Kvβ1, no Kvβ1 oligomers have been detected in the absence of Kv channels. In this scenario, coimmunoprecipitation assays were performed in HEK cells transfected with Kvβ1.1CFP/Kvβ1.1, Kvβ2.1CFP/Kvβ2.1 and Kvβ1.1CFP/Kvβ2.1. Our data showed that in the absence of any Kv α subunit, Kvβ2.1, as well as Kvβ1.1, showed significant homo- and heterocoimmunoprecipitation (Figures 2A–C).

To explore further oligomeric associations, a series of FRET experiments were performed (see representative Kvβ1.1CFP/Kvβ2.1YFP in Figure 2D). Cells were transfected with KvβsCFP (Kvβ1.1CFP, Figure 2Da) and KvβsYFP (Kvβ2.1YFP, Figure 2Db) used as donor and acceptor fluorophores, respectively. Positive colocalization spots (Figure 2Dc) were subject to the acceptor bleach (white square in Figure 2Dd). FRET values confirmed that, similar to the tetrameric Kv1.3 channel (positive control), Kvβ1.1CFP/Kvβ1.1YFP, Kvβ2.1CFP/Kvβ2.1YFP and Kvβ1.1CFP/Kvβ2.1YFP form homo- and hetero-oligomeric complexes (Figure 2E).

### Kvβ Homo- and Hetero-Oligomerization Affinities are Similar

**FIGURE 3:**
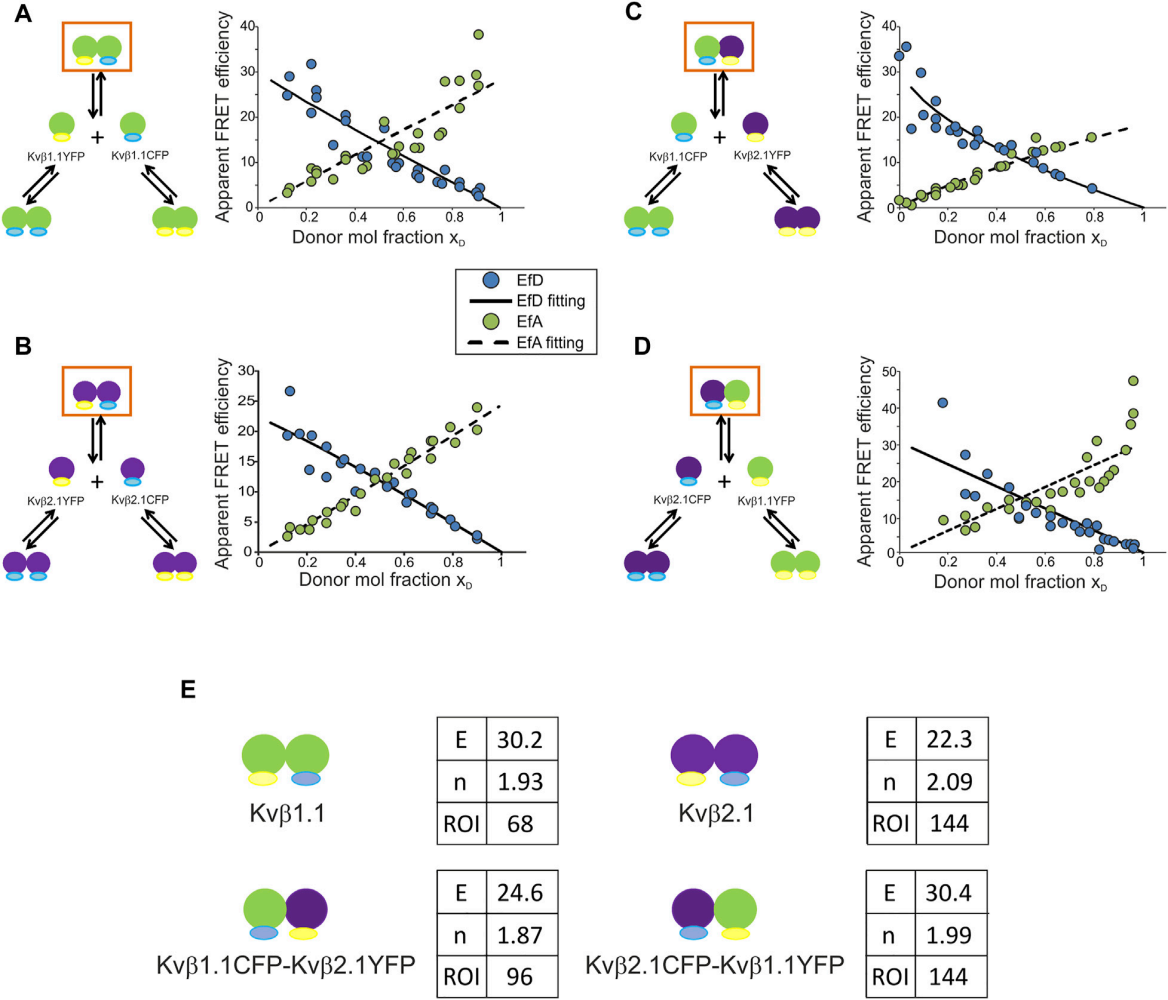
Kvβ1.1 and Kvβ2.1 exhibit high FRET efficiencies for homo- and hetero-oligomerization with a basic unit of two peptides per complex. **(A)** Kvβ1.1CFP/Kvβ1.1YFP. **(B)** Kvβ2.1CFP/Kvβ2.1YFP. **(C)** Kvβ1.1YFP/Kvβ2.1CFP. **(D)** Kvβ1.1CFP/Kvβ2.1YFP. Left panels, schematic representation of possible interactions upon cotransfection. Right panels, plot of Ef_D_ (blue circles), Ef_D_ fitting (solid line), Ef_A_ (green circles), Ef_A_ fitting (dashed line) vs. the donor molar ratio (x_D_). **(E)** Values obtained from lux-FRET experiments performed on Kvβ combinations represented in orange squares in **(A**–**D)**. E, FRET efficiency (%); N, number of units per complex; ROI, number of ROIs analyzed. Green shapes, Kvβ1.1; purple shapes, Kvβ2.1. Yellow represents YFP; blue represents CFP.

Evidence suggests a preferred configuration of Kvβ complexes containing Kvβ2. However, our data indicated that Kvβ1 would also form oligomers in the absence of α-units. To decipher the affinity of the Kvβ complexes, we applied the linear unmixing FRET (lux-FRET) technique, which provides the apparent donor and acceptor FRET efficiencies, stoichiometry and affinity constants of interactions (Wlodarczyk et al., 2008). Experiments were performed in cell suspensions transfected with different donor molar ratios (Figure 3).

Coexpression of Kvβ1.1CFP/Kvβ1.1YFP raised three different complexes: homomeric Kvβ1.1CFP, homomeric Kvβ1.1YFP and Kvβ1.1CFP/Kvβ1.1YFP heteromers. FRET confirmed the formation of heteromeric complexes (orange box in Figure 3A). In this case, lux-FRET values demonstrated an inverse correlation between the FRET efficiency of the donor (Ef_D_) and the donor molar fraction (solid line, Figure 3A). The apparent FRET efficiency of the acceptor (Ef_A_) was the opposite. Thus, the higher the donor molar fraction is, the higher the apparent FRET efficiency (dashed line, Figure 3A). From these data, the FRET efficiency (E) and stoichiometry (N) of the complex were calculated. The FRET efficiency was 30.2%, and the basic unit involved two Kvβ1.1 subunits (*n* = 1.93) (Figure 3E). Kvβ2.1CFP/Kvβ2.1YFP exhibited a similar pattern (Figure 3B), showing 22.3% FRET efficiency and a basic unit of two Kvβ2.1 peptides (*n* = 2.09) (Figure 3E).

We next analyzed Kvβ1.1CFP/Kvβ2.1YFP and reciprocal Kvβ2.1CFP/Kvβ1.1YFP (Figures 3C,D). The Kvβ1.1CFP/Kvβ2.1YFP plot shifted to a lower x_D_ due to a slightly lower expression of Kvβ1.1CFP compared with Kvβ2.1YFP (Figure 3C). In this case, the calculated FRET efficiency was 32.5% with a basic unit of two proteins (*n* = 1.88) per complex (Figure 3E). Kvβ2.1CFP/Kvβ1.1YFP shifted in the opposite x_D_ direction due to the same effect by the lowest Kvβ2.1CFP expression (Figure 3D). In this context, Kvβ2.1 again presented a value of a basic unit of two (*n* = 1.99) and a FRET of 30.4% (Figure 3E).

**FIGURE 4:**
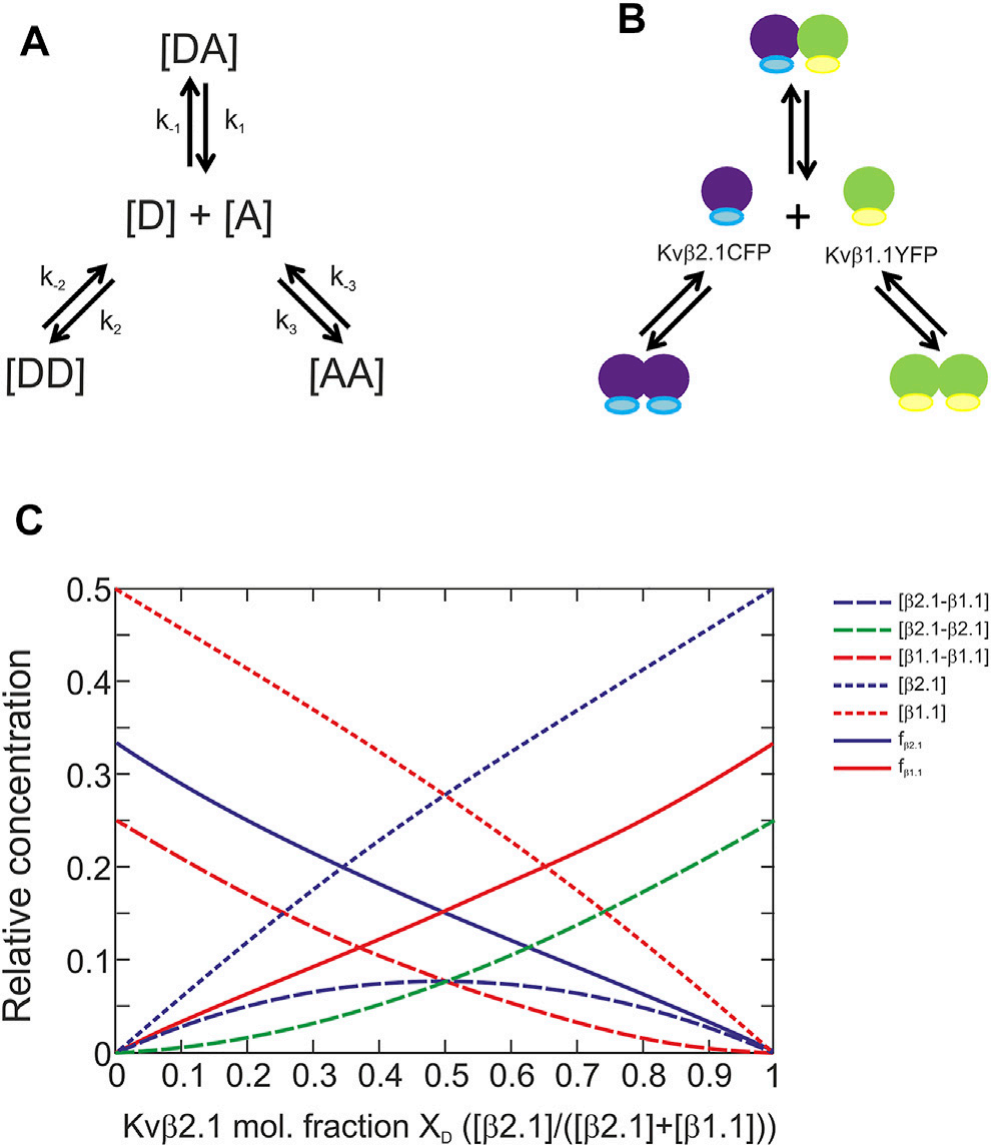
Kvβ1.1 and Kvβ2.1 can form homo- and heteromeric complexes with similar affinities. **(A)** Model of the interaction between donor and acceptor proteins. **(B)** Model of Kvβ1.1 and Kvβ2.1 oligomerization. **(C)**. The calculated plot was based on the formula developed in the MATLAB program to find the affinity constants. Plot of the different parameters [DA], [DD], [AA], [D], [A], f_D_ and f_A_ depending on the donor molar fraction when a model presents the same affinity for forming three different complexes. [β2.1–β1.1], concentration of donor–acceptor complexes. [β2.1–β2.1], concentration of donor–donor complexes. [β1.1–β1.1], concentration of acceptor–acceptor complexes. [β2.1], donor concentration, [β1.1], acceptor concentration. f_β2.1_, apparent donor efficiency. f_β1.1_, apparent acceptor efficiency.

The calculation of the affinity constants was based on the model presented in Figure 4A. The mixture between a donor and an acceptor yields three different complexes. Each complex is formed to a greater or lesser extent depending on their affinity constants (Renner et al., 2012). This model was implemented to solve the Kvβ1.1/Kvβ2.1 affinity (Figure 4B). The system relies on previous evaluation of homomeric forms. Next, the different affinity constants could be defined by using the following formula.

$$\left(-K_{DD}+\sqrt{K_{DD}^{2}+8K_{DD}\left(\left[D_{t}\right]-\left[DA\right]\right)}\right)\times \left(-K_{AA}+\sqrt{K_{AA}^{2}+8K_{AA}\left(\left[A_{t}\right]-\left[DA\right]\right)}\right)=16K_{DA}\left[DA\right]$$

Our abovementioned data were concomitant with the plot in Figure 4C, which suggests that the 3 affinity constants (k) in our model were similar. Thus, unlike 5-HT receptors, the absence of tilted ends in our plots indicated no differences in affinities, and therefore no preferences, between Kvβ1.1 and Kvβ2.1 forming homo- and hetero-oligomers (Renner et al., 2012).

### Kvβ1.1 and Kvβ2.1 Form Tetramers by Dimer Dimerization

**FIGURE 5:**
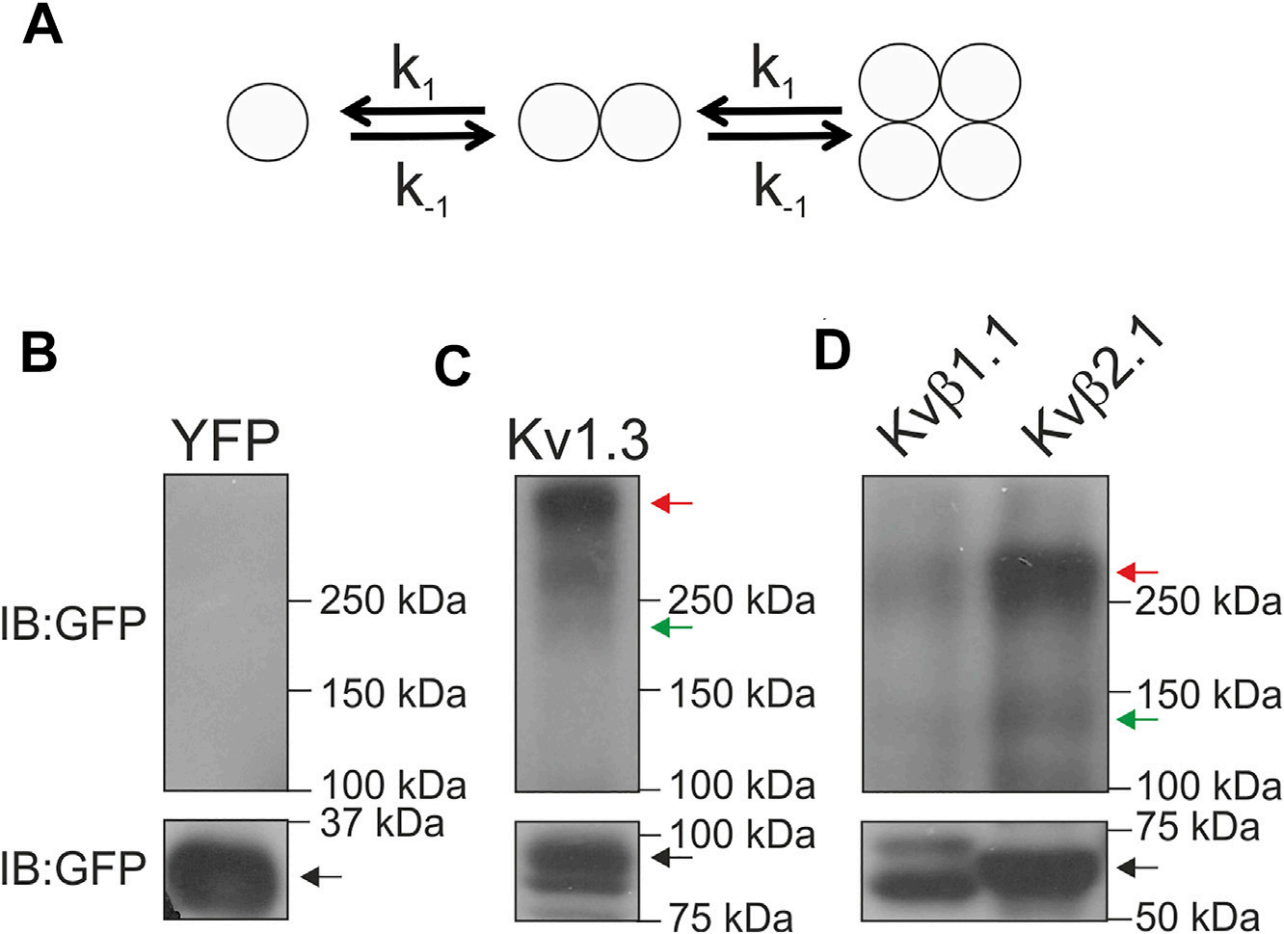
Kvβ1.1 and Kvβ2.1 form dimeric and tetrameric structures. **(A)** Model representing the two putative options for Kvβ oligomerization. Kvβ forms dimers, and Kvβ forms tetrameric structures by association. The affinity constant (k) for every step would be the same in all conditions. **(B**–**D)** HEK 293 cells were transfected with YFP, Kv1.3YFP, Kvβ1.1YFP and Kvβ2.1YFP. Total lysates were analyzed in semidenaturating conditions. **(B)** YFP, **(C)** Kv1.3YFP, **(D)** Kvβ1.1YFP and Kvβ2.1YFP. Immunoblots (IB) were performed with an anti-GFP antibody. Blots were split into two for low- and high-molecular-weight forms for better visualization. The black arrow indicates monomeric forms. The green arrow indicates dimeric forms. The red arrow highlights tetrameric forms.

Our data established that the basic unit for oligomerization was two peptides. This result implies that two different possibilities for the complex dynamics existed: 1) Kvβs form dimers; 2) these dimers oligomerize to form tetrameric structures (Figure 5A). Our results also indicated no trimeric structures (Figure 3E). Because Kvβ2.1 forms tetramers in the absence of the α-subunit, we wondered whether this also applies to the Kvβ1.1 subunit. Semidenaturating gel electrophoresis was implemented in HEK cells transfected with Kvβ1.1CFP and Kvβ2.1CFP (van Huizen et al., 1999). Monomeric structures were detected in all four conditions tested, but unlike YFP-transfected cells (Figure 5B), dimers and tetramers were found in Kv1.3YFP, Kvβ1.1YFP and Kvβ2.1YFP (Figures 5C,D). Kv1.3YFP was used as a control because of its tetrameric architecture. Thus, monomers, some dimers and tetramers were clearly visible (Figure 5C). Similarly, Kvβ1.1 and Kvβ2.1 analysis triggered monomeric, dimeric and tetrameric forms (Figure 5D). Therefore, in agreement with the lux-FRET data, no trimeric complexes were detected. Taken together, our data showed that both Kvβ1.1 and Kvβ2.1 could form tetramers by a dimeric interaction.

### Surface Spatial Localization of Oligomeric Kvβ Compositions

**FIGURE 6:**
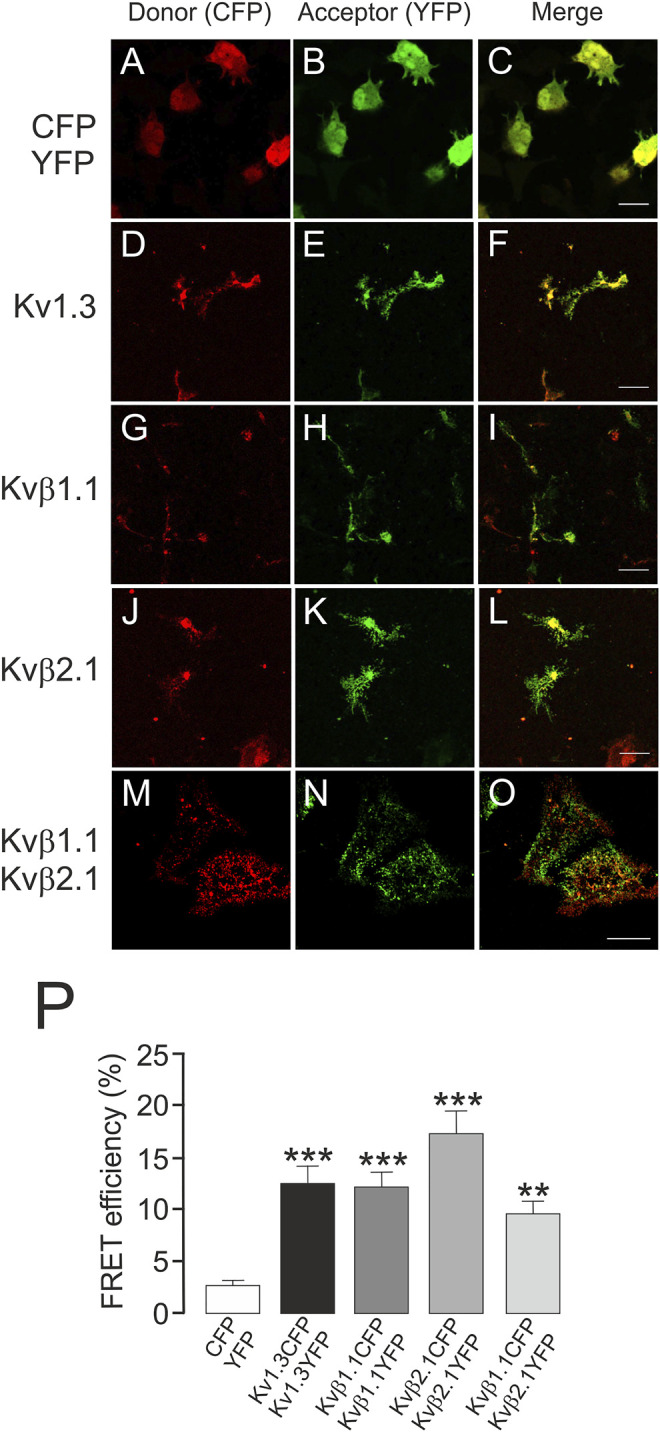
Kvβ1.1 and Kvβ2.1 form heteromeric complexes at the plasma membrane. HEK 293 cells were transfected with Kv1.3YFP-Kv1.3CFP, Kvβ1.1YFP-Kvβ1.1CFP and Kvβ2.1YFP-Kvβ2.1CFP. After transfection, CUPs were purified, and FRET was analyzed. **(A**–**C)** CFP-YFP-transfected cells were used as negative controls. Note that CFP-YFP was analyzed in entire cells. **(D**–**F)** Kv1.3CFP-Kv1.3YFP. **(G**–**I)** Kvβ1.1CFP-Kvβ1.1YFP. **(J**–**L)** Kvβ2.1CFP-Kvβ2.1YFP. **(M**–**O)** Kvβ1.1CFP-Kvβ2.1YFP. Red panels, CFP; green panels, YFP; merged panels, yellow indicates colocalization. **(P)** FRET efficiency (%). **, *p* < 0.01; ***, *p* < 0.001 versus CFP-YFP (Student’s *t* test). Values are the mean of 20–30 cells. Scale bars represent 10 µm.

Kvβ1.1 and Kvβ2.1 target the membrane surface, but only Kvβ2.1 is located in lipid rafts, independent of Kv1.3, in a palmitoylation-dependent manner (Roig et al., 2022). This spatial localization is crucial because Kvβ2.1 clusters at the IS during the immune system response (Beeton et al., 2006; Roig et al., 2022). Therefore, putative oligomeric Kvβ compositions, whose stoichiometry would depend on variable protein expression, could fine-tune leukocyte physiology. In this context, we sought to decipher whether Kvβ subunits target the plasma membrane in the absence of Kvα subunits as homo- or hetero-oligomeric complexes. CUPs were purified from HEK transfected cells, and FRET between Kvβs was analyzed (Figure 6). Only the negative CFP-YFP control was measured in a whole-cell configuration because CFP-YFP is a soluble peptide (Figures 6A–C). The tetrameric Kv1.3CFP/Kv1.3YFP channel was used as a positive control (Figures 6D–F). The FRET efficiency values of Kvβ1.1CFP/Kvβ1.1YFP (Figures 6G–I), Kvβ2.1CFP/Kvβ2.1YFP (Figure 6J–L) and Kvβ1.1CFP/Kvβ2.1YFP (Figures 6M–O) were clearly positive (Figure 6P). These results demonstrated that homo- and hetero-oligomeric Kvβ structures target the plasma membrane.

**FIGURE 7:**
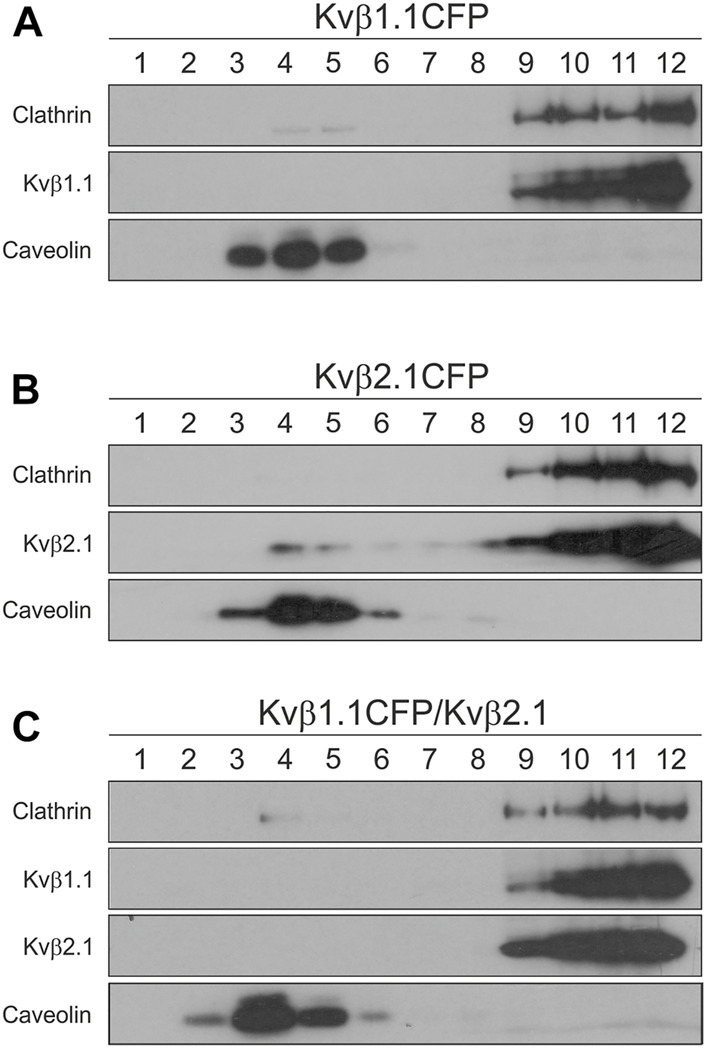
Kvβ1.1 interaction shifts out Kvβ2.1 from lipid raft domains. HEK 293 cells were transfected with Kvβ1.1CFP, Kvβ2.1CFP and Kvβ1.1CFP-Kvβ2.1. Lipid rafts were isolated as described in the methods. One-milliliter fractions were sequentially collected from the top (1, high buoyancy) to the bottom (12, low buoyancy) of the tube. **(A)** Western blotting of lipid raft fractions of Kvβ1.1CFP. **(B)** Western blot analysis of lipid raft fractions of Kvβ2.1. **(C)** Western blotting of lipid raft fractions of Kvβ1.1CFP and Kvβ2.1 cotransfection. While caveolin identifies floating lipid rafts, clathrin indicates nonlipid raft fractions.

Kvβ2.1, but not Kvβ1.1, is locate in lipid rafts (Roig et al., 2022). Because the Kvβ affinity for homo- and heteromultimerization was similar, we investigated whether Kvβ2.1 and Kvβ1.1 would target rafts in a hetero-oligomeric configuration. Low-buoyancy membrane fractions from transfected HEK-293 cells were analyzed. While Kvβ1.1 was not present in raft domains (Figure 7A), Kvβ2.1 exhibited partial localization in these fractions (Figure 7B). Coexpression of both subunits (Kvβ1.1/Kvβ2.1) triggered Kvβ2.1 to no longer traffic to raft microdomains (Figure 7C). Therefore, Kvβ1.1 hetero-oligomerization altered Kvβ2.1 membrane localization in lipid rafts.

## Discussion

**FIGURE 8:**
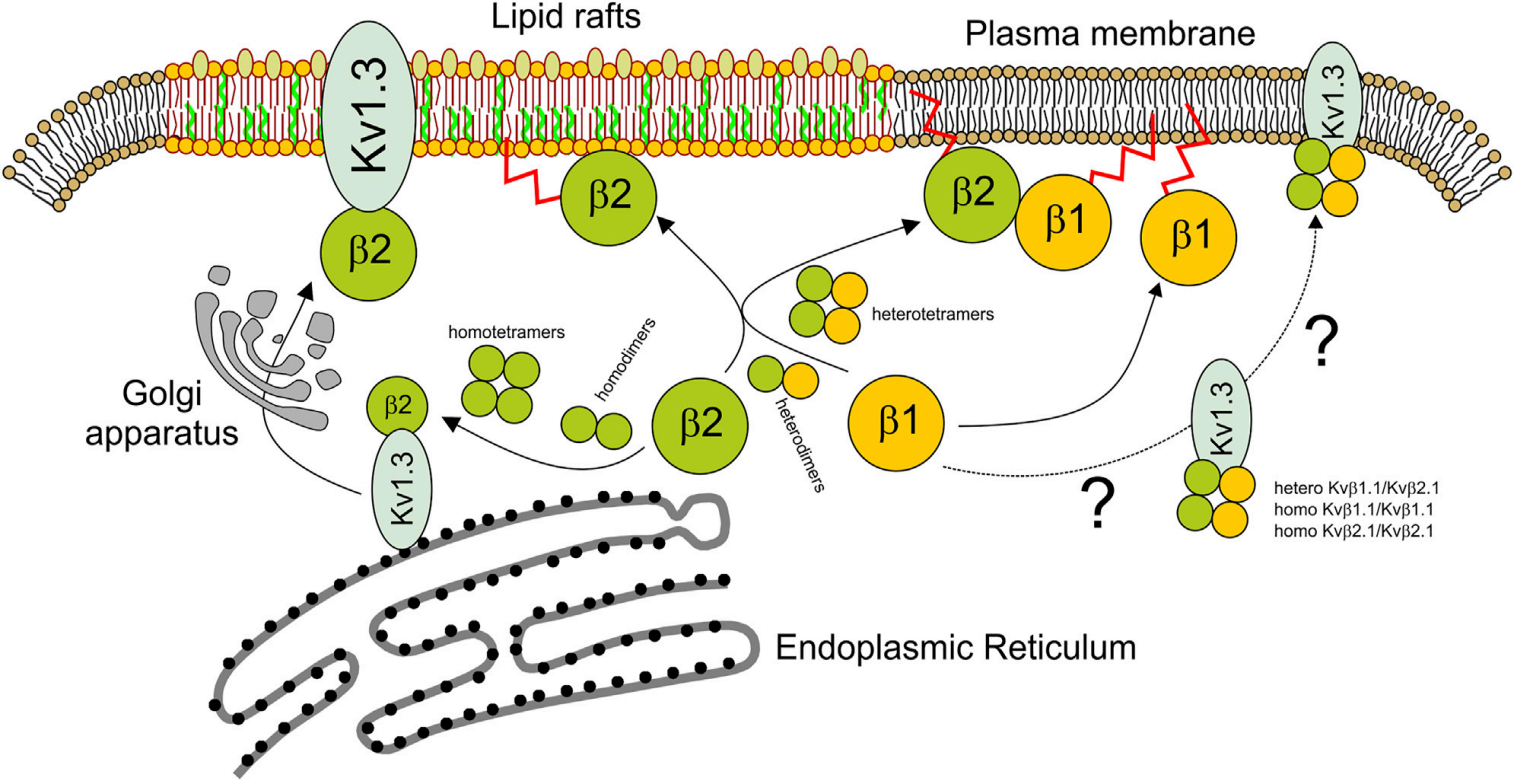
Scheme representing the homo- and heteromeric Kvβ fate. While the tetrameric Kv1.3 channel is assembled early at the ER, soluble Kvβ is synthesized in the cytoplasm. Kvβ would form homo- and heterodimers. Kvβ tetramers would be formed by dimerization of dimers. Kvβ tetramers, especially Kvβ2, interact early with Kv1.3 to target lipid rafts via the Golgi apparatus. The extent to which Kv1.3 associated with Kvβ1.1 or Kvβ1.1/Kvβ2.1 tetramers follows different destinies outside lipid rafts is uncertain. In the absence of a channel, Kvβ2.1 tetramers target lipid rafts in a palmitoylation-dependent manner. However, Kvβ2.1 could also interact with Kvβ1.1, forming heterotetramers. In this context, not only homo-Kvβ1.1 but also Kvβ1.1–Kvβ2.1 heterotetramers would be displaced from lipid rafts targeting other plasma membrane surface locations. By this mechanism, Kvβ1.1 fine-tunes the Kvβ2.1-dependent physiological mechanisms at specific cell surface spots. Blue shape, Kv1.3; green balls, Kvβ2.1; yellow balls, Kvβ1.1. Kvβ palmitoylation (red sparkline).

The physiological function of Kv channels is tightly regulated by regulatory β subunits (Pongs and Schwarz, 2010). The composition and stoichiometry of the α-β complex ultimately determine the kinetics and gating of potassium channels as well as their cellular traffic and distribution (Pongs and Schwarz, 2010). We demonstrated that Kvβ1.1 and Kvβ2.1 form heteromeric complexes. Both peptides present over 85% similarity, and the regions involved in oligomerization are highly conserved. Although the homomeric composition for Kvβ2 was described early (Xu et al., 1998; van Huizen et al., 1999), the tetrameric ability of Kvβ1 subunits is a subject of debate (Accili et al., 1997). The crystal structure of Kvβ2 sustains a tetrameric architecture that was also inferred for Kvβ1 (Gulbis et al., 1999). However, hetero-oligomeric complexes always contain Kvβ2 (Nystoriak et al., 2017). Our work demonstrates that both Kvβs may form homotetramers. The tetramer is generated by dimerization of dimers. Both the homo- and heterotetrameric complexes exhibit similar affinity constants for both Kvβs. Therefore, differential abundance of Kvβs would shape the stoichiometry. In addition, Kvβ2, but not Kvβ1, targets lipid raft microdomains, and the heteromeric composition of the complex impairs the raft location of the Kvβ1/Kvβ2 structure. Given that Kvβ2 clusters at the IS, which concentrates lipid rafts, participating during the immunological response, the Kvβ1 interaction would fine-tune the physiological function by misallocating Kvβ2 from these signaling spots (Figure 8).

Our study also sheds light on the dynamic formation of Kvβ complexes. We found that the tetrameric composition Kvβs follows two sequential steps: 1) dimeric formation and 2) dimer dimerization to form the final tetrameric configuration. Although early evidence suggested trimeric structures (van Huizen et al., 1999), our results can only be fitted to a sequential dimerization of dimers, which would be in agreement with what was described for Kvβ2 homotetramers. Our findings would thereby be concomitant with the oligomerization that Kv α units undergo to form a conducting entity (Hille, 2001). A putative low oligomerization affinity would explain the negative homomeric Kvβ1.1 associations previously documented. In this context, because only Kvβ2 forms tetramers, upon elevated expression, homomeric Kvβ2 complexes displace Kvβ1, impairing its function (Xu and Li, 1997). However, we found that Kvβ1.1 formed oligomers with similar affinity, and the same was true for Kvβ1/Kvβ2 heterotetramerization. In fact, Kvβ1/Kvβ2 heteroligomers are expressed in coronary arterial myocytes, regulating Kv1.5 fine-tuning of the trafficking and membrane localization of the channel (Nystoriak et al., 2017). We confirm previous evidence, but our contribution further shows that Kvβ2.1 and Kvβ1.1 form hetero-oligomers with similar affinities in the absence of the Kv channel. Thus, the unique factor governing multiple stoichiometries would be the differential regulation of both Kvβ peptides. In this scenario, the pattern of Kvβ subunit expression in macrophages depends upon proliferation and the mode of activation (Vicente et al., 2005). Therefore, Kv modulation depends on the final composition of the Kvβ heterotetramer architecture. Several proteins exhibit oligomeric composition control depending on the amount of each partner. For instance, ZIP1/ZIP2/ZIP3 are established hetero- and homodimers depending on the expression level upon different insults (Gong et al., 1999; Croci et al., 2003). In this vein, the heterotetrameric Kv1.3/Kv1.5 channel of professional antigen-presenting cells, such as dendritic cells and macrophages, follow the same fate (Vicente et al., 2006; Villalonga et al., 2007; Vallejo-Gracia et al., 2021). Because two different subunits can govern one unique channel, fine-tuning Kvβ concentrations would trigger a repertoire of functional channels (Pongs and Schwarz, 2010).

Homo- and hetero-oligomers of Kvβ1.1/Kvβ2.1 targeted the membrane surface, but their microdomain localization was different. While Kvβ1.1 is associated with the actin cytoskeleton (Nakahira et al., 1998), Kvβ2.1 partially resides in lipid rafts (Roig et al., 2022). Both Kvβ proteins are palmitoylated, and palmitoylation of Kvβ2 is crucial for its location in these domains (Roig et al., 2022). This is of physiological relevance because Kvβ2 clusters at the IS, which are enriched in lipid rafts, representing an essential hub for signaling during the immune response (Beeton et al., 2006). Kvβ2 is situated in the IS, either modulating Kv1.3 or functioning as a hub for protein–protein interactions. Heterooligomeric interactions between Kvβ1 and Kvβ2 misplace the latter from lipid rafts and impair the function of Kvβ2 in these microdomains. In addition, the presence of Kvβ1 altered the function of Kvβ2 in a concentration-dependent manner. Therefore, the Kvβ1 interaction might fine-tune the Kvβ2-dependent physiological consequences during the immune response. In addition to regulating Kv channels and cluster protein interactions, Kvβs are also AKRs; therefore, redox variations can be sensed (Kilfoil et al., 2013). The different distribution of Kvβ throughout the cell surface would provide a differential redox sensitivity in different microdomains. Moreover, the diverse affinity for NADPH determines differential spatial triggers. Kvβ2 forms part of the signaling complex, which interacts with CD4, Kv1.3, ZIP1/2 and PSD proteins and clusters at the immunological synapse in human T cells (Beeton et al., 2006; Roig et al., 2022). In addition, within these locations, Kvβ2 is regulated by PKC, p56lck and other signaling kinases (Kwak et al., 1999; Wang et al., 2004; Kim et al., 2005; Roepke et al., 2007; Ishii et al., 2013). Therefore, any spatial alteration in the localization of Kvβ2, as well as changes in Kvβ2-dependent enzymatic functions, such as modulating the Kv1.3 channelosome, surely would have essential consequences for leukocyte physiology.

## Data Availability

The original contributions presented in the study are included in the article/Supplementary Material, further inquiries can be directed to the corresponding author.
