# Supplementary figures and images for: Oligomerization and Spatial Distribution of Kvβ1.1 and Kvβ2.1 Regulatory Subunits
Source: Front Physiol. 2022 Jun 17;13:930769. doi: 10.3389/fphys.2022.930769 (PMC9247503; doi:10.3389/fphys.2022.930769)

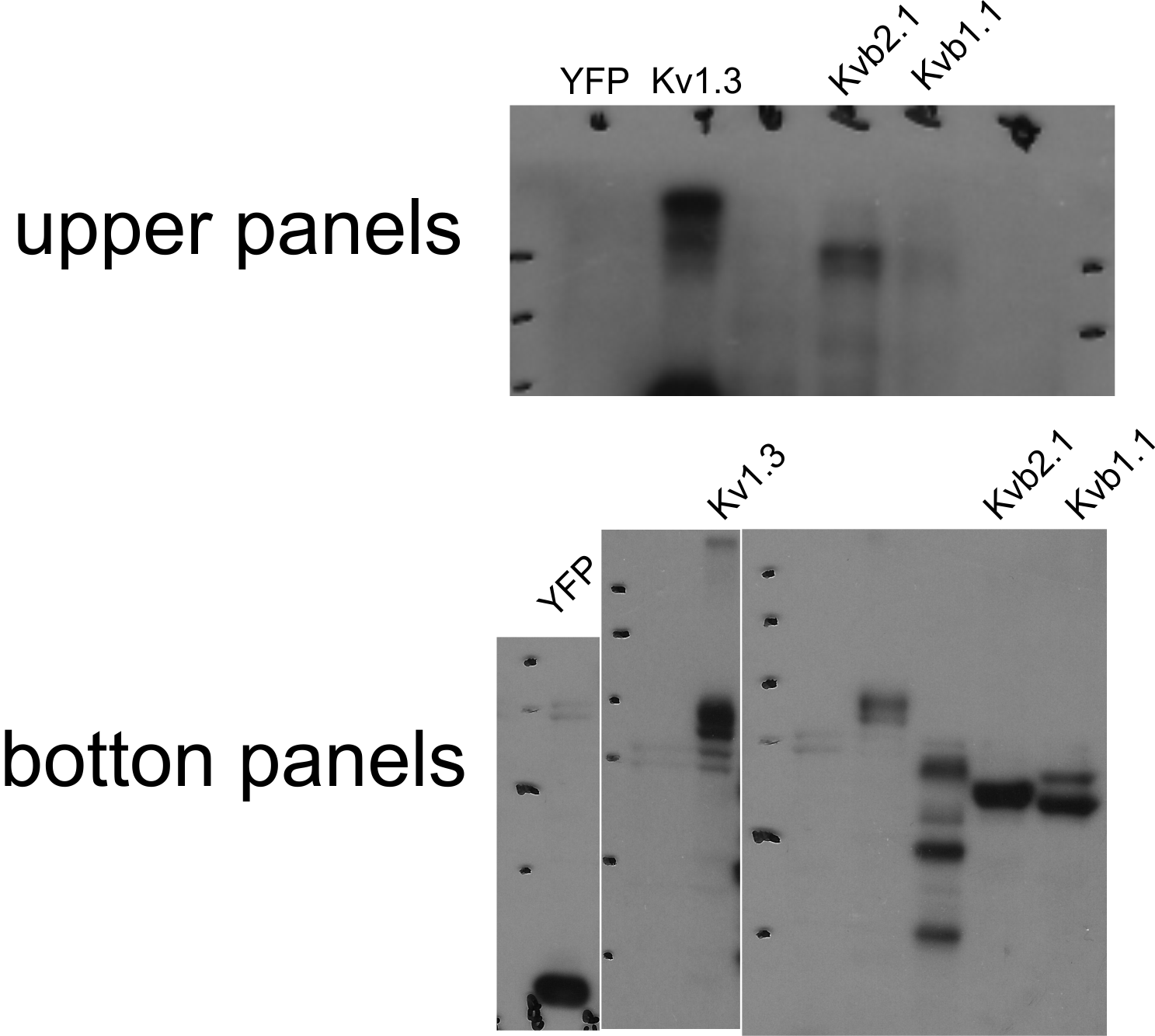

Supplement: Supplementary file 1 [file Image3.TIF]

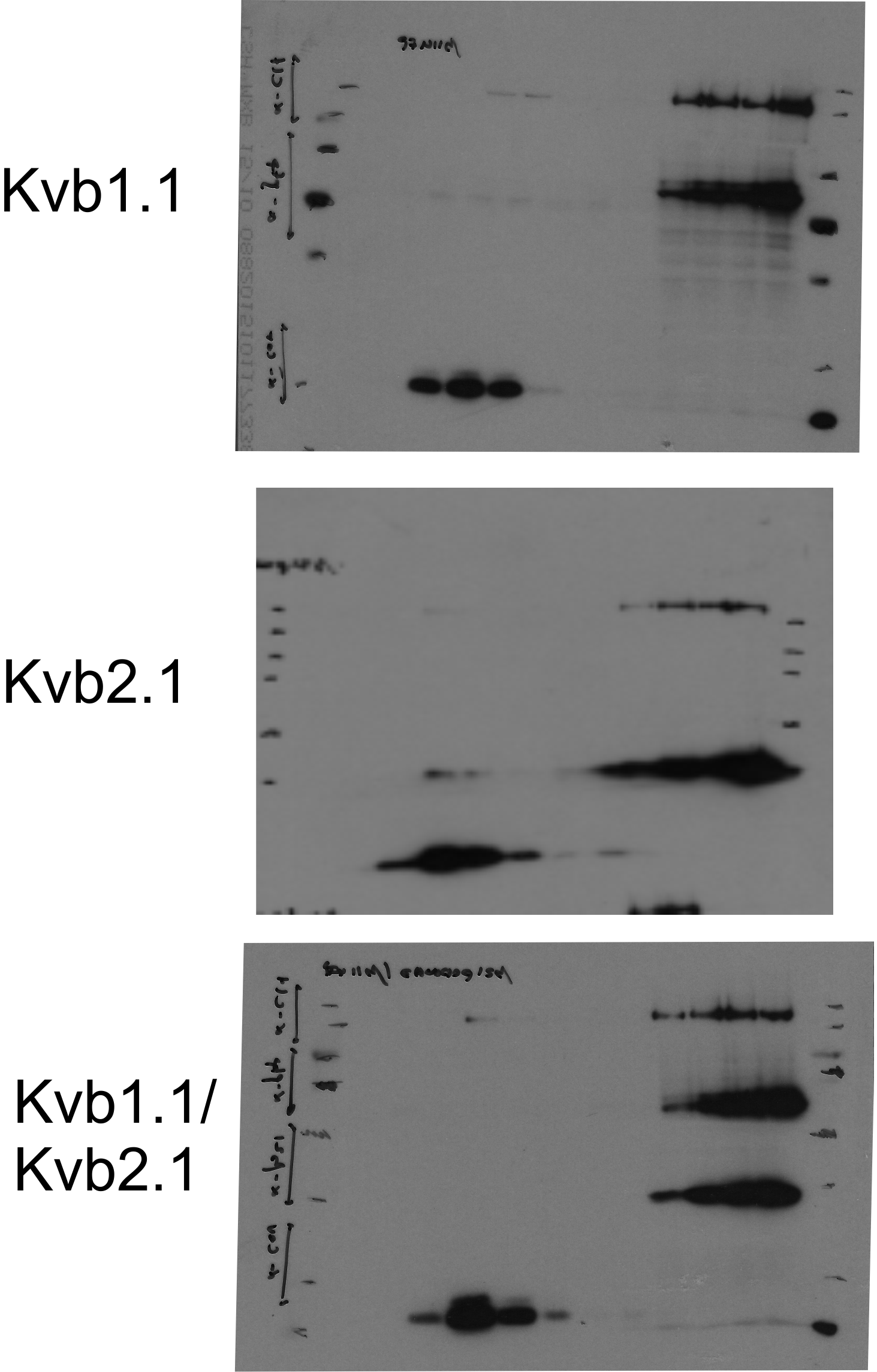

Supplement: Supplementary file 2 [file Image4.TIF]

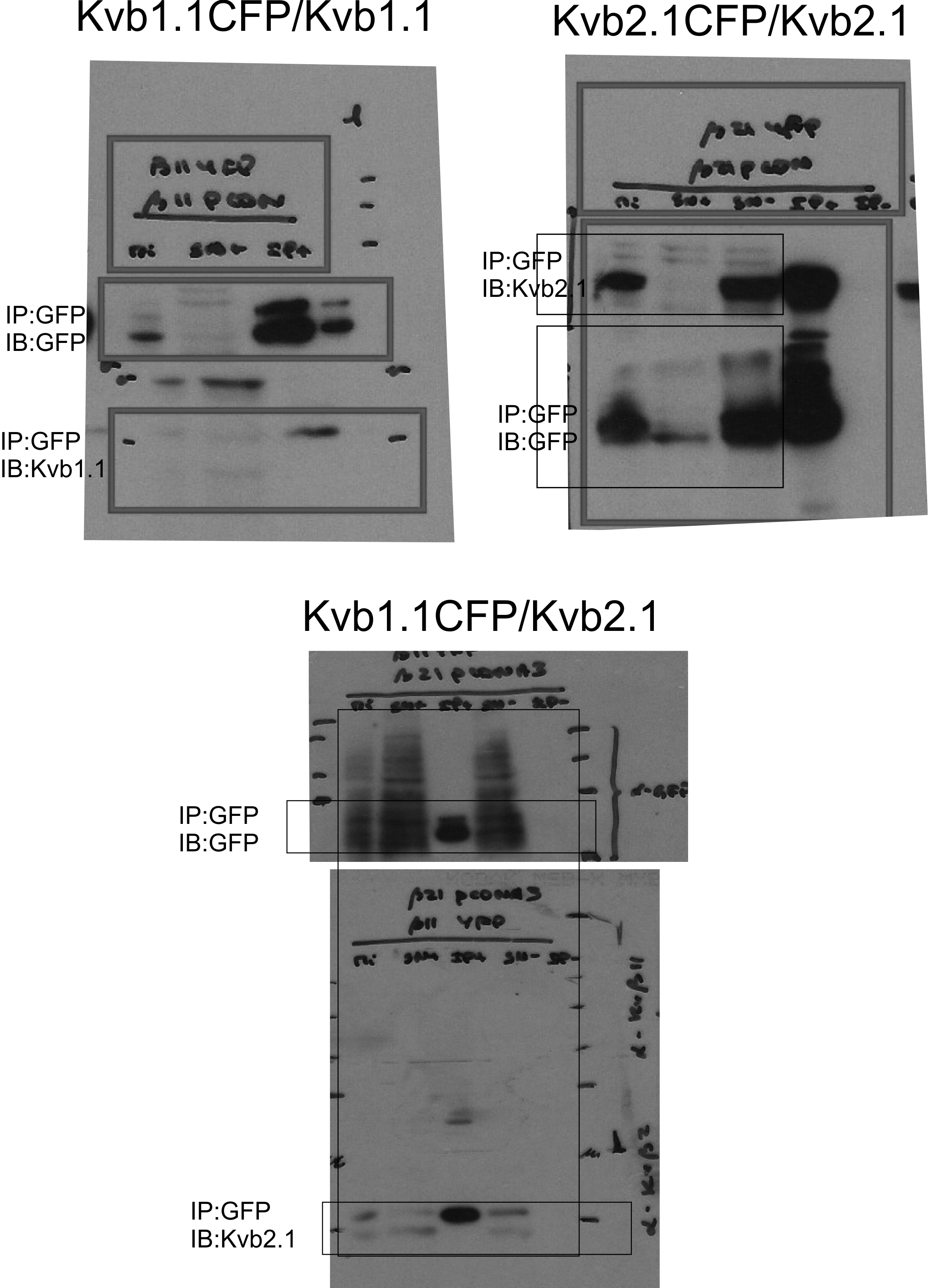

Supplement: Supplementary file 3 [file Image2.TIF]

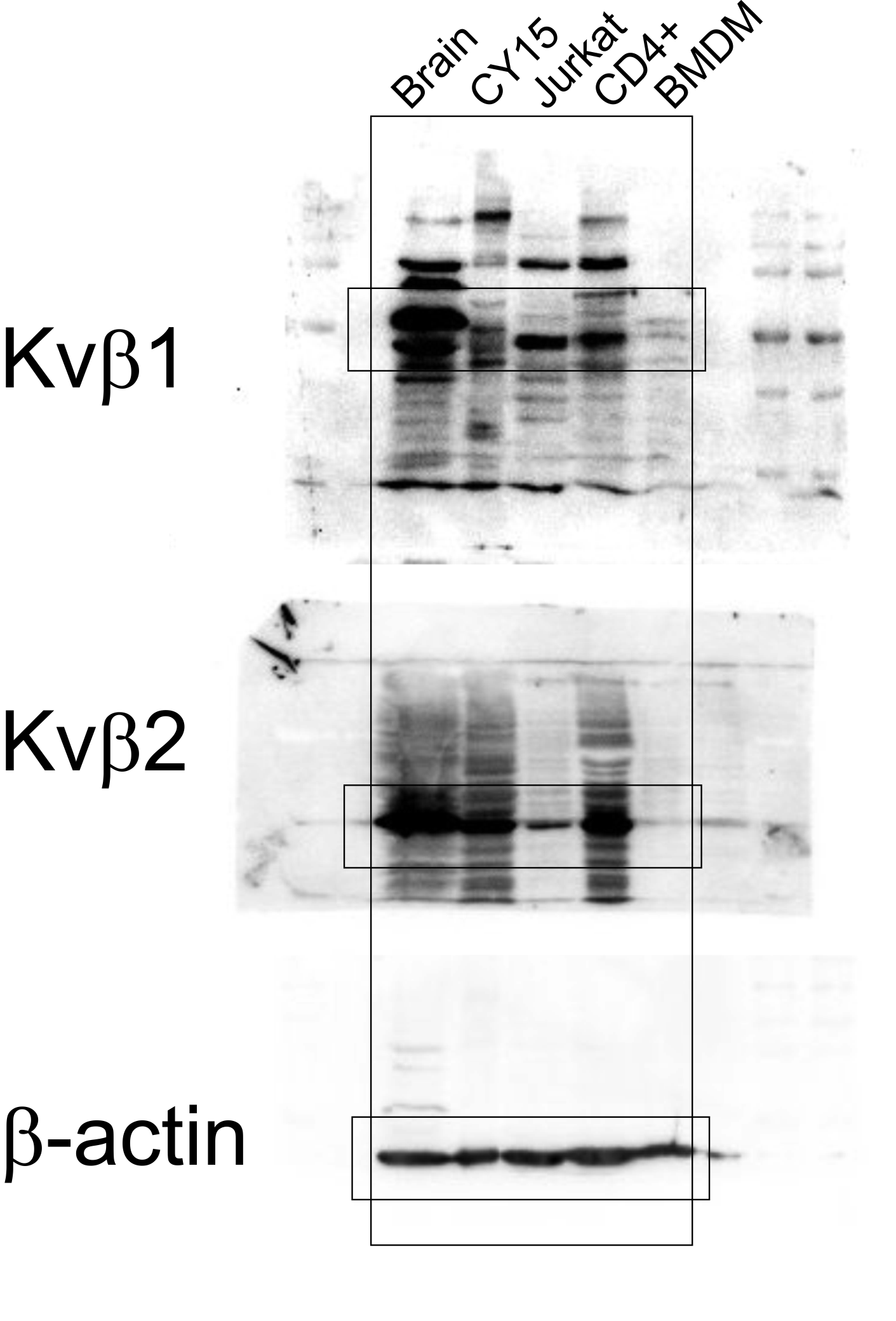

Supplement: Supplementary file 4 [file Image1.TIF]
